# Effect of Magnesium Salt Whiskers on the Mechanical Properties of Phosphogypsum Building Blocks

**DOI:** 10.3390/ma18051152

**Published:** 2025-03-04

**Authors:** Jiang He, Maiping Yan, Kaizhi Gu, Xiangming Li, He Wei

**Affiliations:** 1 National & Local Joint Engineering Laboratory for Technology of Advanced Metallic Solidification Forming and Equipment, Kunming 650093, China; hj05220601@163.com (J.H.); 20222230205@stu.kust.edu.cn (M.Y.); gkzkust@163.com (K.G.); 2 Faculty of Material Science and Engineering, Kunming University of Science and Technology, Kunming 650093, China; 3 Faculty of Highway and Construction Engineering, Yunnan Communications Vocational and Technical College, Kunming 650500, China

**Keywords:** solid waste, phosphogypsum, magnesium whiskers, DFT

## Abstract

Phosphogypsum (CaSO_4_⋅2H_2_O), as an industrial by-product widely used in the field of building materials, has garnered considerable attention for its mechanical properties. This study explores the effect of magnesium (Mg) doping on phosphogypsum’s (CaSO_4_⋅2H_2_O) mechanical properties. Using first principles, it found that Mg doping increases the bulk, shear, and Young’s moduli of phosphogypsum from 42.52445, 19.76419, and 51.33892 GPa to 48.22389, 22.98504, and 59.36072 GPa, respectively, and hardness from 3.18363 GPa to 3.6273 GPa. It also determined the interface binding stability with magnesium salts, ranking the stability as CaSO_4_·2H_2_O/Mg(OH)_2_ > CaSO_4_⋅2H_2_O/MgSO_4_ > CaSO_4_⋅2H_2_O/MgCl_2_. Tests showed Mg-salt-doped phosphogypsum’s compressive and flexural strength increased by 14.72% and 20.61%, respectively, enhancing its value in construction. This finding holds significant implications for enhancing the application value of phosphogypsum in the construction materials sector.

## 1. Introduction

Phosphogypsum, a by-product of wet-process phosphoric acid production, is primarily formed by the reaction of sulfuric acid with phosphate rock [1,2,3,4,5,6]. For every ton of wet-process phosphoric acid produced, approximately 4 to 6 tons of phosphogypsum are generated, contributing to a substantial amount of industrial solid waste. Globally, billions of tons of phosphogypsum are produced annually [7,8,9]. This material is composed mainly of hydrated calcium sulfate (CaSO_4_⋅2H_2_O), along with small quantities of impurities, such as phosphorus, fluorine, organic substances, oxides, heavy metals, and radioactive materials [10,11,12,13]. The prolonged storage of phosphogypsum poses significant environmental risks, making its treatment and reuse critical issues in environmental and resource management [1,2,14,15,16].

Due to these concerns, and given the annual large-scale production of phosphogypsum, finding sustainable methods for its storage, treatment, and utilization has become an essential task in environmental management. Countries like China, India, Japan, the Philippines, Russia, and the United States have started using phosphogypsum as a raw material in building materials production, such as cement, bricks, and gypsum boards, thanks to its primary component, hydrated calcium sulfate [13,17,18,19,20,21,22]. However, for broader application in construction, phosphogypsum must exhibit enhanced strength to meet the requirements for durability, safety, and performance in various applications [23,24,25,26,27].

In light of phosphogypsum’s growing use in building materials, numerous studies have focused on modifying its properties to increase its utility in construction. For example, Nizevičienė [28] and colleagues found that adding 5% hydronium jarosite to phosphogypsum, followed by 2 min of ultrasonic treatment, could increase its compressive strength by 35%. Similarly, Wu [29] and others demonstrated that the mechanical and waterproofing properties of phosphogypsum blocks could be optimized under specific conditions, such as a compaction pressure of 300 MPa, a curing time of 3 days, a water content of 5%, and a 1% content of iron and aluminum elements. Furthermore, incorporating fibers into gypsum has been shown to significantly enhance its strength, toughness, and durability, leading to the development of fiber-reinforced gypsum-based composite materials [24,30,31,32]. Yet, research on magnesium salt whisker-reinforced phosphogypsum composite materials remains relatively scarce.

To address this gap, our study focuses on the mechanical properties of Mg^2+^-doped hydrated calcium sulfate (CaSO_4_⋅2H_2_O). We investigated the interface binding properties between phosphogypsum and various magnesium salts (Mg(OH)_2_, MgCl_2_, MgSO_4_) to identify the salt with the best interface binding performance. A 10 Å vacuum layer was introduced in the interface structure to minimize interactions among surrounding atoms. To ensure computational accuracy, a total energy tolerance of 2 × 10^−5^ eV/atom and a maximum stress tolerance of 0.05 were employed.

## 2. Calculation and Experimental Methods

### 2.1. Calculation Methods

In this study, first-principles methods based on density functional theory (DFT) were employed, with corrections for electron exchange–correlation energies performed using the Generalized Gradient Approximation (GGA). For structural optimization calculations, both GGA and the Perdew Becke Ernzerhof (PBE) functionals were utilized. The valence electrons for H, O, S, Ca, and Mg were treated as 1s^1^, 2s^2^2p^4^, 3s^2^3p^4^, 3s^2^3p^6^4s^2^, and 2s^2^2p63s^2^. Throughout the computational process, the self-consistent field (SCF) iterative method and reciprocal space ultrasoft pseudopotentials were employed. After convergence testing, the plane wave cutoff energy was set to 650 eV. For the Brillouin zone (BZ) sampling, k-point grids of 3 × 1 × 2, 3 × 2 × 2, and 4 × 4 × 2 for crystal structures; 2 × 1 × 1, 3 × 2 × 1, and 4 × 2 × 1 for surface structures; and 3 × 1 × 1 for interface structures were used. The convergence criterion for SCF was set to 1 × 10^−6^ eV/atom. In this work, the BFGS algorithm was employed for the optimization of atomic structures, with convergence criteria set as follows: forces acting on individual atoms converged to 0.01 eV/Å, stress deviations were less than 0.02 GPa, and lattice parameter deviations were below 0.0005 Å.

### 2.2. Computational Model

**Figure 1:**
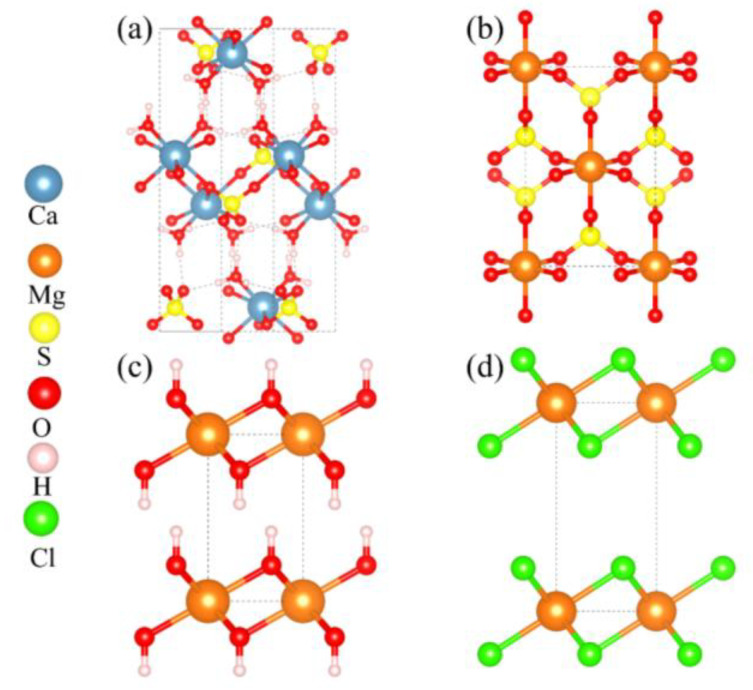
Crystal Structure Models. (**a**) CaSO_4_⋅2H2O; (**b**) MgSO_4_; (**c**) Mg(OH)_2_; (**d**) MgCl_2_.

The calculations presented aimed to evaluate the mechanical properties of Mg^2+^-doped hydrated calcium sulfate (CaSO_4_·2H_2_O), along with exploring the interfacial binding properties between various magnesium salts and hydrated calcium sulfate. The crystal structures analyzed in this study are illustrated in Figure 1. Specifically, the crystal structure of CaSO_4_·2H_2_O is characterized as belonging to the monoclinic crystal system, consisting of a total of 48 atoms. In contrast, MgSO_4_ crystallizes in the orthorhombic crystal system, comprising 24 atoms. Additionally, Mg(OH)_2_ is noted for its trigonal crystal structure with 5 atoms, while MgCl_2_ also presents a trigonal crystal structure but with 3 atoms.

### 2.3. Experimental Method

**Figure 2:**
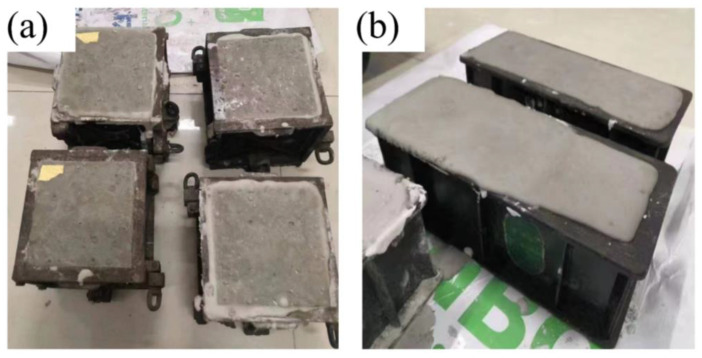
Phosphogypsum Mixing Process and Test Molds. (**a**) Phosphogypsum Mixing; (**b**) Cubic Test Mold.

In this experiment, a water drill mixer was used to stir the phosphogypsum, and the stirred phosphogypsum was then placed into cubic and rectangular molds. This allowed the phosphogypsum blocks to naturally air dry within the molds. The phosphogypsum block molding process and the mold are shown in in Figure 2. The composition of the phosphogypsum blocks is provided in Table 1.

Compressive strength tests were conducted on phosphogypsum bricks before and after the introduction of magnesium salt whiskers using a hydraulic press. To ensure uniform loading conditions, steel plates were placed above and below each specimen during the tests. Flexural strength tests on phosphogypsum bricks with and without magnesium salt whiskers were carried out using a flexural testing machine. Additionally, scanning electron microscopy (SEM) was utilized to observe the microstructure before and after the introduction of magnesium-doped phosphogypsum. Due to the poor electrical conductivity of phosphogypsum, gold sputtering was applied to the samples to enhance the visibility of the microstructure.

## 3. Results and Discussion

### 3.1. Crystal Structure Stability

The binding strength of CaSO_4_⋅2H_2_O and CaSO_4_⋅2H_2_O doped with Mg after mixing can be determined by calculating the mixing enthalpy [33,34,35]. The formula for calculating the mixing enthalpy [34,36] is as follows:

(1) $$E_{\mathrm{coh}}({\mathrm{CaSO}}_{4}\text{·}2H_{2}O)=\left[E_{\mathrm{tot}}\left({\mathrm{CaSO}}_{4}\text{·}2H_{2}O\right)-aE_{\mathrm{Ca}}-bE_{S}-cE_{O}-dE_{H}\right]/\left(a+b+c+d\right)$$

(2) $$E_{\mathrm{coh}}[{\mathrm{Ca}(\mathrm{Mg})\mathrm{SO}}_{4}\text{·}2H_{2}O]=\left[E_{\mathrm{tot}}[{\mathrm{Ca}(\mathrm{Mg})\mathrm{SO}}_{4}\text{·}2H_{2}O]-aE_{\mathrm{Ca}}-b-bE_{S}-cE_{O}-dE_{H}\right]/\left(a+b+c+d\right)$$

In the formula, $E_{\mathrm{coh}}({\mathrm{CaSO}}_{4}\text{·}2H_{2}O)$ represents the total energy of CaSO_4_·2H_2_O, while $E_{\mathrm{coh}}[{\mathrm{Ca}(\mathrm{Mg})\mathrm{SO}}_{4}\text{·}2H_{2}O]$ represents the total energy of CaSO_4_·2H_2_O doped with Mg after mixing. The variables E_Ca_, E_S_, E_O_, E_H_, and E_Mg_, respectively, denote the single-atom energies of Ca, S, O, H, and Mg atoms.

**Figure 3:**
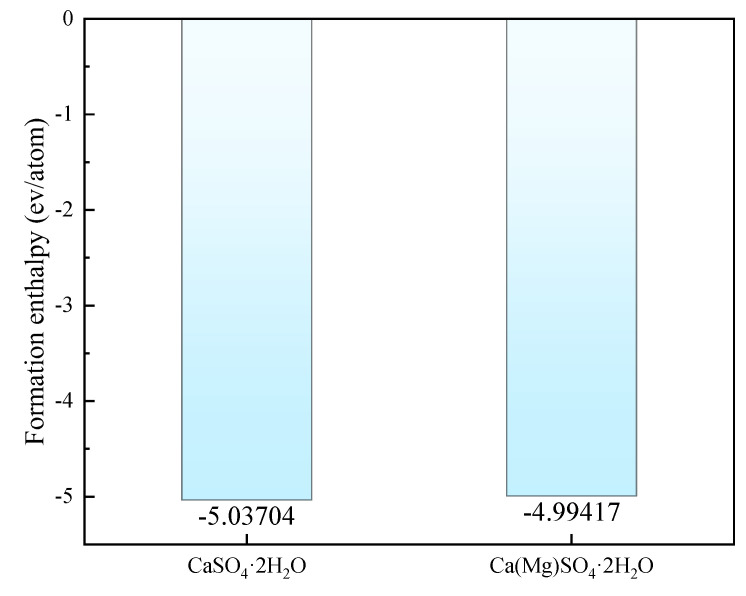
Mixing Enthalpy of CaSO_4_·2H_2_O and Ca(Mg)SO_4_·2H_2_O.

As shown in Figure 3, the mixed enthalpies of pure CaSO_4_⋅2H_2_O and Mg-doped CaSO4⋅2H2O were compared. The results indicate that the mixed enthalpy slightly increases after Mg doping, reaching −4.99417 eV, but still remains below zero. The stability of crystal structures can be determined by whether the mixed enthalpy is less than zero: if the mixed enthalpy is greater than zero, the crystal structure is unstable; if the mixed enthalpy is less than zero, the crystal structure is stable. Therefore, the system with Mg doping, having a mixed enthalpy that is still below zero, can be considered stable.

### 3.2. Influence of Mg Doping on the Mechanical Properties of CaSO_4_·2H_2_O

#### 3.2.1. Elastic Modulus and Mechanical Performance Stability

The mechanical properties of phosphogypsum (CaSO_4_·2H_2_O) are crucial performance indicators to determine whether it meets the usage requirements. These properties can be assessed by calculating the elastic constants of phosphogypsum using first-principles calculations. In this study, first-principles calculations were employed to determine the volume modulus, shear modulus, Young’s modulus, and Poisson’s ratio of phosphogypsum before and after Mg doping. The volume modulus and shear modulus of Mg-doped CaSO_4_·2H_2_O (monoclinic crystal) before and after doping were calculated using the Voigt–Reuss–Hill approximation method. The average of the results obtained from the Voigt and Reuss methods is considered the true elastic modulus of the polycrystalline material [37]. The calculation formulas are as follows [38,39]:

(3) $$B_{V}=\left[(C_{11}+C_{22}+C_{33})+(C_{12}+C_{13}+C_{23})\right]/9$$

(4) $$B_{R}={\left[S_{11}+S_{22}+S_{33}+2(S_{12}+S_{13}+S_{23})\right]}^{-1}$$

(5) $$G_{V}=\left[\left(C_{11}+C_{22}+C_{33}\right)-\left(C_{12}+C_{13}+C_{23}\right)+3(C_{44}+C_{55}+C_{66}\right)/15$$

(6) $$G_{R}=15[4\left(S_{11}+S_{22}+S_{33}\right)-4\left(S_{12}+S_{13}+S_{13}\right)+3\left(S_{44}+S_{55}+S_{66}\right)]$$

In the formulas, [C_ij_] and [S_ij_] represent the elastic stiffness matrix and elastic compliance matrix, respectively.

Based on the Voigt–Reuss–Hill approximation, the Hill values for the volume modulus and shear modulus are defined by equations. Furthermore, Young’s modulus (E) and Poisson’s ratio (ν) can be determined from the volume modulus and shear modulus [40].

(7) $$B_{H}=\left(B_{V}+B_{R}\right)/2$$

(8) $$B_{H}=\left(B_{V}+B_{R}\right)/2$$

(9) E=9BG/(3B+G)

(10) v=(3B−2G)/2(3B+G)

**Figure 4:**
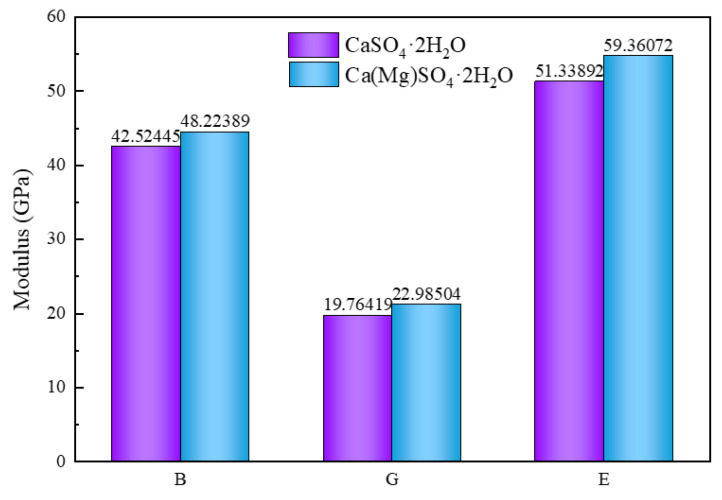
Elastic Modulus of CaSO_4_·2H_2_O and Ca(Mg)SO_4_·2H_2_O.

The volume modulus, shear modulus, and Young’s modulus of CaSO_4_·2H_2_O before and after Mg doping are presented in Figure 4. The volume modulus, shear modulus, and Young’s modulus of CaSO_4_·2H_2_O are 42.52445, 19.76419, and 51.33892 GPa, respectively. After Mg doping, these values increase to 48.22389, 22.98504, and 59.36072 GPa. Following Mg doping, the volume modulus, shear modulus, and Young’s modulus exhibit an increase of 13.43803%, 16.29639%, and 15.62518%, respectively.

**Figure 5:**
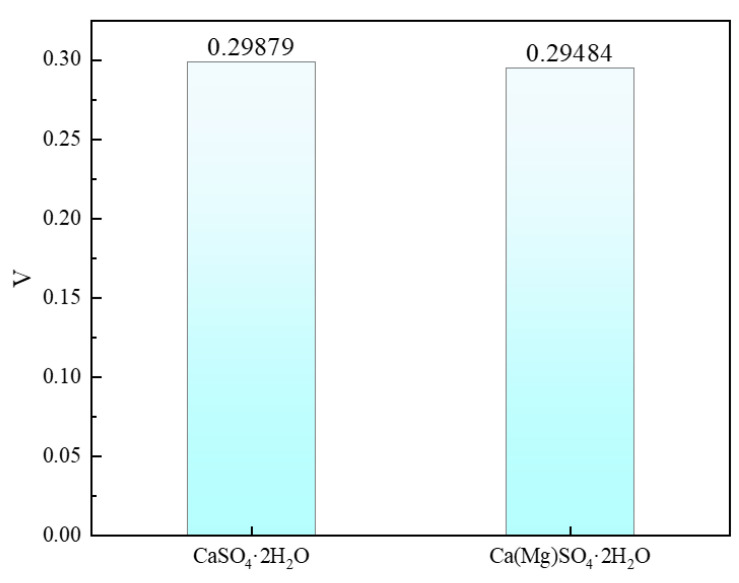
Poisson’s Ratio of CaSO_4_·2H_2_O and Ca(Mg)SO_4_·2H_2_O.

Poisson’s ratio is one of the important parameters for assessing material properties and can to some extent indicate the ductility of a material [41]. The calculation results are shown in Figure 5. Before Mg doping, the Poisson’s ratio of CaSO_4_·2H_2_O is 0.29879, and after doping, it is slightly reduced to 0.29484.

To assess the mechanical performance stability of CaSO_4_·2H_2_O before and after Mg doping, the Born criterion is used based on the elastic constants C_ij_. Both CaSO_4_·2H_2_O before and after Mg doping belong to the monoclinic crystal system. C_ij_ is shown in Table 2. The Born criterion for the monoclinic crystal system is as follows [42]:

(11) $$C_{ii}>0(i=1-6)$$

(12) $$C_{11}+C_{22}+C_{33}+2C_{12}+2C_{13}+2C_{23}>0$$

(13) $$C_{33}C_{55}-C_{35}^{2}>0$$

(14) $$C_{44}C_{66}-C_{46}^{2}>0$$

(15) $$C_{22}+C_{33}>2C_{23}$$

(16) $$C_{22}\left(C_{33}C_{55}-C_{35}^{2}\right)+2C_{23}C_{25}C_{35}-C_{23}^{2}C_{55}-C_{25}^{2}C_{33}>0$$

(17) $$\begin{array}{l} 2[C_{15}C_{25}\left(C_{33}C_{12}-C_{13}C_{23}\right)+C_{15}C_{35}\left(C_{22}C_{13}-C_{12}C_{23}\right)\\ \;+\;C_{25}C_{35}\left(C_{11}C_{23}\right.]\left.\left.-C_{12}C_{13}\right)\right]\\ \;-\;[C_{15}^{2}\left(C_{22}C_{33}-C_{23}^{2}\right)+C_{25}^{2}\left(C_{11}C_{33}-C_{13}^{2}\right)\\ \;+\;C_{35}^{2}\left(C_{11}C_{22}-C_{12}^{2}\right)]\\ \;+\;C_{55}\left(C_{11}C_{22}C_{33}-C_{11}C_{23}^{2}-C_{22}C_{13}^{2}-C_{33}C_{12}^{2}+2C_{12}C_{13}C_{23}\right)\\ \;>\;0 \end{array}$$

According to the results of the elastic matrix calculations, both pure CaSO_4_⋅2H_2_O and Mg-doped CaSO_4_⋅2H_2_O have their elastic constants C_ij_ greater than zero. Additionally, both materials meet the criteria of Equations (12)–(17), conforming to the Born stability criteria for monoclinic crystal systems. Therefore, the mechanical properties of these materials demonstrate stability, both before and after doping with Mg.

#### 3.2.2. Hardness

Hardness is a crucial parameter for describing the mechanical characteristics of materials. It is typically understood as a material’s ability to resist elastic and plastic deformation. Materials with high hardness can withstand significant pressure without undergoing deformation, while materials with low hardness are more prone to deformation. Currently, many research scholars use first-principles calculations to predict the hardness values of materials, and it has been demonstrated that first-principles predictions of material hardness are quite accurate [43]:

(18) $$H_{V}=0.92{\left(\frac{B}{G}\right)}^{-1.137}G^{0.708}$$

**Figure 6:**
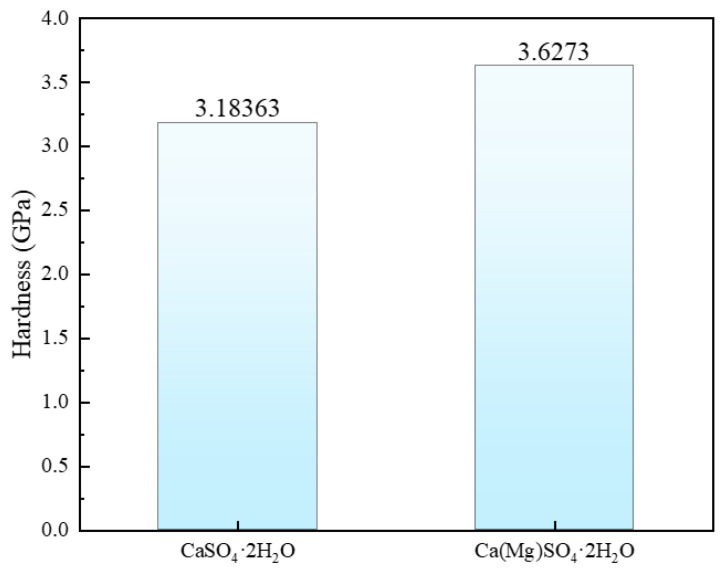
Hardness of CaSO_4_·2H_2_O and Ca(Mg)SO_4_·2H_2_O.

In this study, we used Formula (18) to determine the hardness of CaSO_4_·2H_2_O before and after Mg doping. The hardness values are presented in Figure 6. The hardness of CaSO_4_·2H_2_O is 3.18363 GPa, while after Mg doping, the hardness of Ca(Mg)SO_4_·2H_2_O reaches 3.6273 GPa, representing a 13.9359% increase in hardness. Material hardness is typically associated with compressive performance, where higher hardness indicates the ability to withstand greater pressure without damage. The increase in hardness of CaSO_4_·2H_2_O after Mg doping suggests that Mg doping enhances the compressive performance of phosphogypsum.

### 3.3. The Impact of Mg Doping on the Electronic Properties of CaSO_4_·2H_2_O

To elucidate the mechanism of Mg doping’s impact on the electronic properties of CaSO_4_·2H_2_O, the density of states (DOS) for CaSO_4_·2H_2_O and Ca(Mg)SO_4_·2H_2_O was calculated.

**Figure 7:**
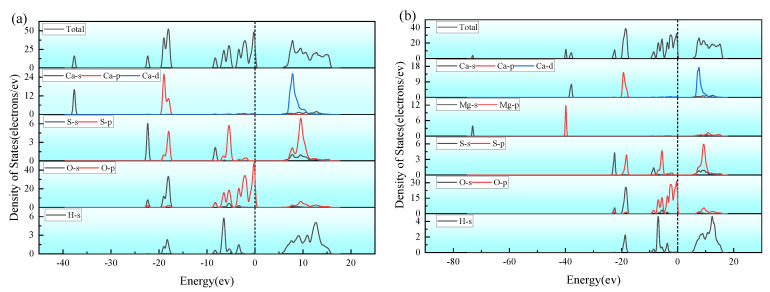
Density of States (DOS) of CaSO_4_·2H_2_O and Ca(Mg)SO_4_·2H_2_O; (**a**) CaSO_4_⋅2H_2_O; (**b**) Ca(Mg)SO_4_⋅2H_2_O.

Figure 7 depicts the Density of States (DOS) of CaSO_4_·2H_2_O and Ca(Mg)SO_4_·2H_2_O. From the Figure, it can be observed that the main contribution to the Fermi level comes from the O-P orbitals. Moreover, within the range of −9.5 to 0 eV, there is hybridization between the O-s orbitals, S-p orbitals, and H-S orbitals, resulting in sp orbital hybridization [44]. This leads to the formation of H-S bonds, O-S bonds, and H-O bonds. In the −20 to −17 eV range, there is hybridization between H-s orbitals and Ca-p orbitals, resulting in the formation of H-Ca bonds. Analysis of the partial density of states (P-DOS) graph reveals that there is partial overlap between O-s and O-p orbitals, both of which contribute to the total state density, indicating the possibility of O-O bond formation. After Mg doping, there is no apparent orbital hybridization observed in the P-DOS graph, suggesting that covalent bonds related to Mg may not have formed.

The results of the Mulliken Population Analysis are presented in Table 3. In the analysis of CaSO_4_·2H_2_O and the Mg-doped Ca(Mg)SO_4_·2H_2_O, several key changes are observed. Firstly, after Mg doping, the average population values of H-H and H-O bonds in Ca(Mg)SO_4_·2H_2_O are lower than those in CaSO_4_·2H_2_O, indicating that Mg doping has affected the electron density distribution in these bonds. Specifically, the average population values of H-H and H-O bonds exhibit different characteristics in both materials: the average population value of H-H bonds is less than 0, while the average population value of H-O bonds is greater than 0, suggesting that H-O bonds have more electron accumulation than H-H bonds. Regarding bond lengths, Mg doping leads to a decrease in the average bond length of H-H bonds and an increase in the average bond length of H-O bonds in Ca(Mg)SO_4_·2H_2_O, reflecting that H-H bonds become tighter while H-O bonds become relatively loose. Additionally, Mg doping increases the number of H-H and H-O bonds in Ca(Mg)SO_4_·2H_2_O, further demonstrating the significant impact of Mg doping on these bonds. On the other hand, the number of O-O bonds in Ca(Mg)SO_4_·2H_2_O is significantly greater than in CaSO_4_·2H_2_O, and Mg doping reduces both the average population value and the average bond length of O-O bonds. This indicates that Mg doping strengthens the interaction between O-O bonds. Furthermore, Mg substitution doping reduces the number of O-S bonds and decreases the number of H-Ca and O-Ca bonds related to Ca, but increases the number of H-Mg and O-Mg bonds related to Mg. These observations suggest that Mg doping significantly alters the electronic structure and bonding characteristics of CaSO_4_·2H_2_O, which may be related to the higher elastic modulus of Ca(Mg)SO_4_·2H_2_O compared to CaSO_4_·2H_2_O after Mg doping.

### 3.4. Interfacial Properties of CaSO_4_·2H_2_O with Magnesium Salts (Mg(OH)_2_, MgCl_2_, MgSO_4_)

#### 3.4.1. Surface Energy of CaSO_4_·2H_2_O with MgSO_4_, Mg(OH)_2_, and MgCl_2_

To establish interfaces between CaSO_4_·2H_2_O/MgSO_4_, CaSO_4_·2H_2_O/Mg(OH)_2_, and CaSO_4_·2H_2_O/MgCl_2_, five low-index surfaces of CaSO_4_·2H_2_O with Mg(OH)_2_, MgCl_2_, and MgSO_4_ were cut. The surface energies of CaSO_4_·2H_2_O and the five surfaces with Mg(OH)_2_, MgCl_2_, and MgSO_4_, which are (100), (110), (111), (210), and (211) respectively, were calculated. The magnitude of the surface energy indicates the stability of the surface; the lower the surface energy, the more stable the surface [45]. The formation of an interface is usually by the surface with the lowest surface energy. To construct the interface between CaSO_4_·2H_2_O and different magnesium salts (Mg(OH)_2_, MgCl_2_, MgSO_4_) and to verify the interfacial binding properties between CaSO_4_·2H_2_O and various magnesium salts, surfaces for constructing the interface were selected. The formula for calculating surface energy is as follows [46]:

(19) $$\delta_{\mathrm{surf}\;}=\frac{E_{\mathrm{slab}\;}-\left[\frac{N_{\mathrm{slab}\;}}{N_{\mathrm{bulk}\;}}\right]E_{\mathrm{bulk}\;}}{{2A}_{\mathrm{slab}}}$$

**Figure 8:**
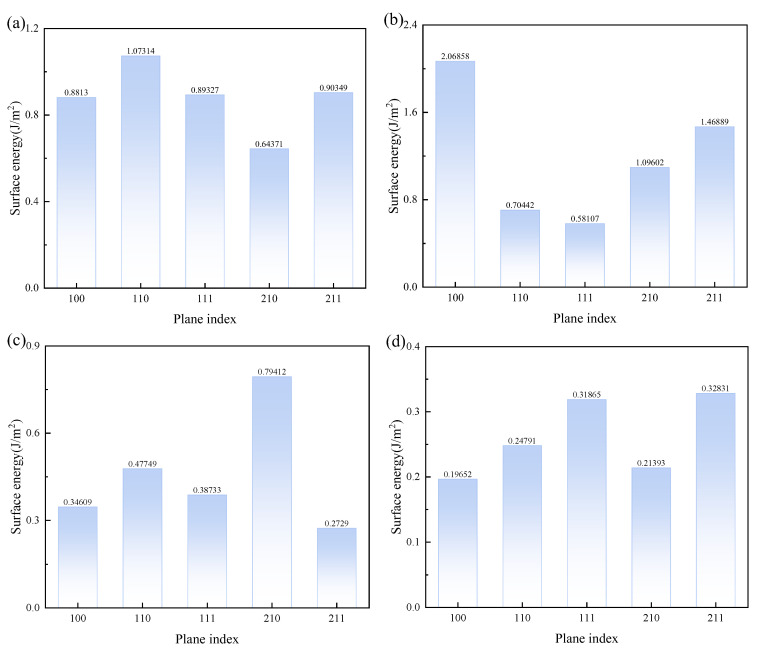
Surface Energy of CaSO_4_·2H_2_O with MgSO_4_, Mg(OH)_2_, MgCl_2_. (**a**) CaSO_4_⋅2H_2_O; (**b**) MgSO_4_; (**c**) Mg(OH)_2_; (**d**) MgCl_2_.

In this context, $E_{\mathrm{slab}}$ represents the total energy (in eV) of the surface with an added vacuum layer, while $E_{\mathrm{bulk}}$ is the total energy (in eV) of the bulk material. $N_{\mathrm{slab}\;}$ and $N_{\mathrm{bulk}\;}$ denote the number of atoms in the surface and in the bulk, respectively. $A_{\mathrm{slab}\;}$ refers to the surface area of the cut face. The surface energy results for CaSO_4_·2H_2_O with MgSO_4_, Mg(OH)_2_, and MgCl_2_ are shown in Figure 8. The surface of CaSO_4_·2H_2_O with the lowest surface energy is the 210 surface, for MgSO_4_ it is the 111 surface, and for Mg(OH)_2_, the surface with the lowest energy is 211, which is also the lowest for Mg(OH)_2_.

#### 3.4.2. Interface Stability of CaSO_4_·2H_2_O/MgSO_4_, CaSO_4_·2H_2_O/Mg(OH)_2_, and CaSO_4_·2H_2_O/MgCl_2_

**Figure 9:**
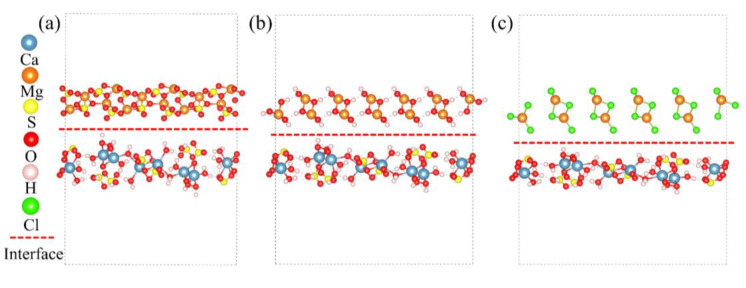
Interface Model. (**a**) CaSO_4_⋅2H_2_O/MgSO_4_; (**b**) CaSO_4_⋅2H_2_O/Mg(OH)_2_; (**c**) CaSO_4_⋅2H_2_O/MgCl_2_.

Based on the calculated surface energy results, interface models of CaSO_4_·2H_2_O/MgSO_4_, CaSO_4_·2H_2_O/Mg(OH)_2_, and CaSO_4_·2H_2_O/MgCl_2_ were constructed. The constructed interface models are shown in Figure 9. Subsequently, the interfacial energy and adhesion work of these interfaces were calculated.

Interfacial energy and adhesion work can both be used to assess the stability of an interface; the lower the interfacial energy, the more stable the interface [47]. The formula for calculating interfacial energy is as follows [48]:

(20) $$\begin{array}{l} \gamma_{int}=\frac{E_{{CaSO}_{4}\cdot 2H_{2}O/{MgSO}_{4}}-N_{{CaSO}_{4}\cdot 2H_{2}O}E_{{CaSO}_{4}\cdot 2H_{2}O}^{bulk}-N_{{MgSO}_{4}}E_{{MgSO}_{4}}^{bulk}}{A_{{CaSO}_{4}\cdot 2H_{2}O/{MgSO}_{4}}}\\ \;-\delta_{{CaSO}_{4}\cdot 2H_{2}O}-\delta_{{MgSO}_{4}} \end{array}$$

(21) $$\begin{array}{l} \gamma_{int}=\frac{E_{{CaSO}_{4}\cdot 2H_{2}O/{Mg(OH)}_{2}}-N_{{CaSO}_{4}\cdot 2H_{2}O}E_{{CaSO}_{4}\cdot 2H_{2}O}^{bulk}-N_{{Mg(OH)}_{2}}E_{{Mg(OH)}_{2}}^{bulk}}{A_{{CaSO}_{4}\cdot 2H_{2}O/{Mg(OH)}_{2}}}\\ \;-\delta_{{CaSO}_{4}\cdot 2H_{2}O}-\delta_{{Mg(OH)}_{2}} \end{array}$$

(22) $$\begin{array}{l} \gamma_{int}=\frac{E_{{CaSO}_{4}\cdot 2H_{2}O/{MgCl}_{2}}-N_{{CaSO}_{4}\cdot 2H_{2}O}E_{{CaSO}_{4}\cdot 2H_{2}O}^{bulk}-N_{{MgCl}_{2}}E_{{MgcL}_{2}}^{bulk}}{A_{{CaSO}_{4}\cdot 2H_{2}O/{MgCl}_{2}}}-\delta_{{CaSO}_{4}\cdot 2H_{2}O}\\ \;-\delta_{{MgCl}_{2}} \end{array}$$

(23) $$W_{ad}=\frac{E_{{CaSO}_{4}\cdot 2H_{2}O}^{slab}+E_{{MgSO}_{4}}^{slab}-E_{{CaSO}_{4}\cdot 2H_{2}O/{MgSO}_{4}}}{A_{{CaSO}_{4}\cdot 2H_{2}O/{MgSO}_{4}}}$$

(24) $$W_{ad}=\frac{E_{{CaSO}_{4}\cdot 2H_{2}O}^{slab}+E_{{Mg(OH)}_{2}}^{slab}-E_{{CaSO}_{4}\cdot 2H_{2}O/{Mg(OH)}_{2}}}{A_{{CaSO}_{4}\cdot 2H_{2}O/{Mg(OH)}_{2}}}$$

(25) $$W_{ad}=\frac{E_{{CaSO}_{4}\cdot 2H_{2}O}^{slab}+E_{{MgCl}_{2}}^{slab}-E_{{CaSO}_{4}\cdot 2H_{2}O/{MgCl}_{2}}}{A_{{CaSO}_{4}\cdot 2H_{2}O/{MgCl}_{2}}}$$

In the formula, $E_{{\mathrm{CaSO}}_{4}\text{⋅}2H_{2}O/{\mathrm{MgSO}}_{4}}$, $E_{{\mathrm{CaSO}}_{4}\text{⋅}2H_{2}O/{\mathrm{Mg}(\mathrm{OH})}_{2}}$, and $E_{{CaSO}_{4}\cdot 2H_{2}O/{MgCl}_{2}}$ represent the total energies of the CaSO_4_·2H_2_O/MgSO_4_, CaSO_4_·2H_2_O/Mg(OH)_2_, and CaSO_4_·2H_2_O/MgCl_2_ interfaces, respectively. $N_{{\mathrm{CaSO}}_{4}\text{⋅}2H_{2}O}$, $N_{{\mathrm{MgSO}}_{4}}$, $N_{{\mathrm{Mg}(\mathrm{OH})}_{2}}$, and $N_{{MgCl}_{2}}$ are the numbers of CaSO_4_·2H_2_O, MgSO_4_, Mg(OH)_2_, and MgCl_2_ molecules in the corresponding interfaces, respectively. $\delta_{{\mathrm{CaSO}}_{4}\text{⋅}2H_{2}O}$, $\delta_{{\mathrm{MgSO}}_{4}}$, $\delta_{{\mathrm{Mg}(\mathrm{OH})}_{2}}$, and $\delta_{{\mathrm{MgCl}}_{2}}$ correspond to the surface energies of CaSO_4_·2H_2_O, MgSO_4_, Mg(OH)_2_, and MgCl_2_, respectively. $E_{{CaSO}_{4}\cdot 2H_{2}O}^{slab}$, $E_{{MgSO}_{4}}^{slab}$, $E_{{Mg(OH)}_{2}}^{slab}$, and $E_{{MgCl}_{2}}^{slab}$ correspond to the total energies of the surfaces of CaSO_4_·2H_2_O, MgSO_4_, Mg(OH)_2_, and MgCl_2_, respectively. $A_{{\mathrm{CaSO}}_{4}\text{⋅}2H_{2}O/{\mathrm{MgSO}}_{4}}$, $A_{{\mathrm{CaSO}}_{4}\text{⋅}2H_{2}O/{\mathrm{Mg}(\mathrm{OH})}_{2}}$, and $A_{{\mathrm{CaSO}}_{4}\text{⋅}2H_{2}O/{\mathrm{MgCl}}_{2}}$ are the surface areas of the CaSO_4_·2H_2_O/MgSO_4_, CaSO_4_·2H_2_O/Mg(OH)_2_, and CaSO_4_·2H_2_O/MgCl_2_ interfaces, respectively.

**Figure 10:**
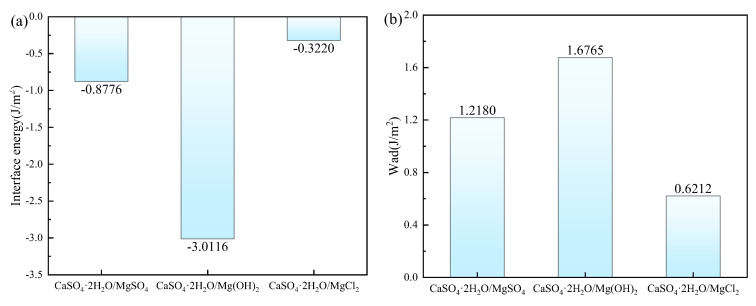
The Interface Energy and Adhesive Work. (**a**) Interface Energy; (**b**) Adhesive Work.

The interfacial energy and adhesive work for CaSO_4_⋅2H_2_O/MgSO_4_, CaSO_4_⋅2H_2_O/Mg(OH)_2_, and CaSO_4_⋅2H_2_O/MgCl_2_ are shown in Figure 10. The order of interfacial energy magnitude is CaSO_4_⋅2H_2_O/MgCl_2_ > CaSO_4_⋅2H_2_O/MgSO_4_ > CaSO_4_⋅2H_2_O/Mg(OH)_2_, while the order of adhesive work magnitude is CaSO_4_⋅2H_2_O/Mg(OH)_2_ > CaSO_4_⋅2H_2_O/MgSO_4_ > CaSO_4_⋅2H_2_O/MgCl_2_. Since smaller interfacial energy corresponds to larger adhesive work, the interface becomes more stable. Therefore, the ranking order for interface binding stability is CaSO_4_⋅2H_2_O/Mg(OH)_2_ > CaSO_4_⋅2H_2_O/MgSO_4_ > CaSO_4_⋅2H_2_O/MgCl_2_.

#### 3.4.3. Analysis of Interface Electronic Structure

To investigate the interface electronic structure of CaSO_4_⋅2H_2_O/MgSO_4_, CaSO_4_⋅2H_2_O/Mg(OH)_2_, and CaSO_4_⋅2H_2_O/MgCl_2_, the interface differential charge density for CaSO_4_⋅2H_2_O/MgSO_4_, CaSO_4_⋅2H_2_O/Mg(OH)_2_, and CaSO_4_⋅2H_2_O/MgCl_2_ was computed.

**Figure 11:**
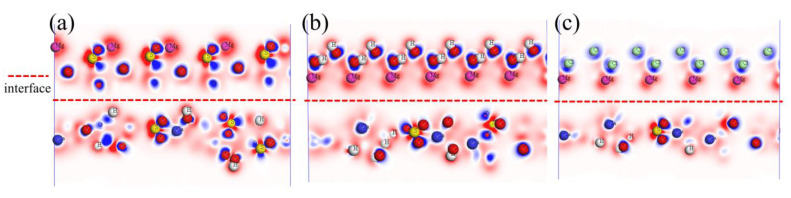
Interface Differential Charge. (**a**) CaSO_4_⋅2H_2_O/MgSO_4_; (**b**) CaSO_4_⋅2H_2_O/Mg(OH)_2_; (**c**) CaSO_4_⋅2H_2_O/MgCl_2_.

Differential charge density directly reflects the essential characteristics of chemical bonding within a crystal structure. The results are shown in Figure 11, where the blue regions represent gained electrons, and the red regions represent lost electrons. In the interface structures of CaSO_4_⋅2H_2_O/MgSO_4_ and CaSO_4_⋅2H_2_O/Mg(OH)_2_, oxygen (O) atoms gain electrons, while sulfur (S) atoms, hydrogen (H) atoms, calcium (Ca) atoms, and magnesium (Mg) atoms lose electrons. In the interface structure of CaSO_4_⋅2H_2_O/MgCl_2_, oxygen (O) atoms and chlorine (Cl) atoms gain electrons, while hydrogen (H) atoms, calcium (Ca) atoms, and magnesium (Mg) atoms lose electrons.

### 3.5. Experimental Results

#### 3.5.1. Impact of Magnesium Salts on the Mechanical Properties of Bricks

Based on the calculation results, Mg(OH)_2_ whiskers were chosen to be added to pure phosphogypsum. Subsequently, the compressive strength and flexural strength of the phosphogypsum masonry blocks were tested before and after the addition of magnesium salt whiskers. Compressive strength, a key indicator of material strength in construction, refers to the ability of a material to withstand pressure without breaking. Flexural strength refers to the material’s resistance to bending or twisting forces. Both of these properties are crucial in architectural design as they directly impact the stability, safety, and longevity of structures [49,50,51].

**Figure 12:**
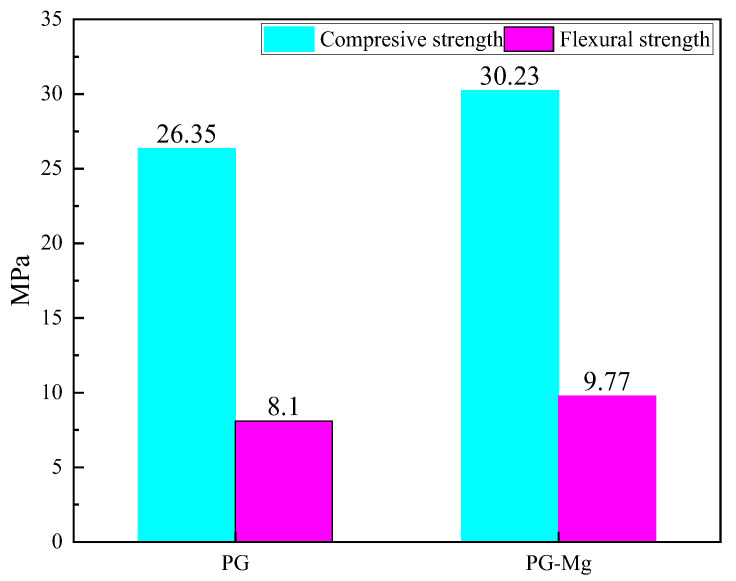
Compressive Strength and Flexural Strength of Phosphogypsum Masonry Block Specimens.

As shown in Figure 12, the compressive strength of pure phosphogypsum blocks is 26.35 MPa. After doping with Mg salt, the compressive strength increases to 30.23 MPa, an improvement of 14.72%. In addition, the bending strength with magnesium salt whiskers added reaches 9.77 MPa, compared to 8.10 MPa for pure phosphogypsum, an increase of 20.61%. Therefore, it is evident that the addition of magnesium salt whiskers significantly enhances both the compressive and bending strengths of the material, with a particularly notable improvement in bending strength.

#### 3.5.2. Microscopic Morphology

In order to analyze the reasons for the improvement in the compressive and flexural performance of phosphogypsum blocks when magnesium salts are added, SEM characterization analysis was conducted on the fracture surfaces of the blocks before and after the addition of magnesium salts.

**Figure 13:**
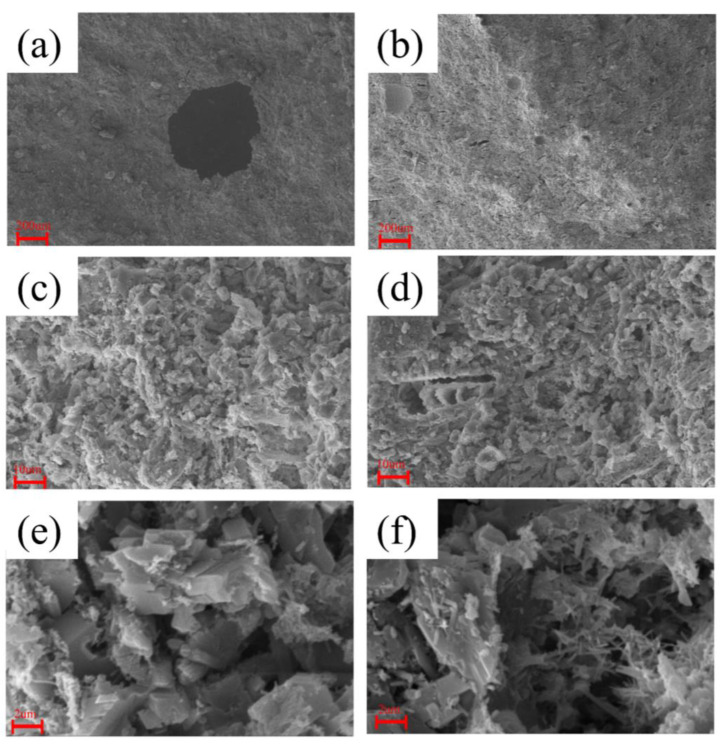
SEM Scanning of Phosphogypsum Specimens. (**a**) Microstructure of Phosphogypsum at 50× Magnification; (**b**) Microstructure of Magnesium Salt Whisker-Doped at 50× Magnification; (**c**) Microstructure of Phosphogypsum at 1000× Magnification; (**d**) Microstructure of Magnesium Salt Whisker-Doped at 1000× Magnification; (**e**) Microstructure of Phosphogypsum at 6000× Magnification; (**f**) Microstructure of Magnesium Salt Whisker-Doped at 6000× Magnification.

The characterization results are shown in Figure 13. Figure 13a,c,e depict the morphology of pure phosphogypsum, while Figure 13b,d,f show the morphology after doping with magnesium salt whiskers. From Figure 13a,f, it is observed that the micro-morphology of the composite material generally presents a needle-like and columnar crystal structure with overlapping crystals. According to Figure 13a,b, the particles with added magnesium salt whiskers are finer and do not exhibit large pores, which significantly affect the cutting performance of the blocks. Furthermore, as shown in Figure 13f, after the addition of magnesium salt whiskers, the microscopic images of the whiskers added to the matrix are displayed. This indicates that the whiskers can increase the number of effective contact positions between crystals in the composite material, and they act to fill the pores. When subjected to force, the effective contact points between the whiskers and the dihydrate gypsum crystals can disperse stress, reducing stress concentration. In addition, there are some fibrous whiskers that might act similarly to fibers in the phosphogypsum blocks, potentially serving as connectors for the particles. This is similar to the role of fibers, which can bridge cracks and pores [52]. Due to these effects, magnesium salt whiskers enhance the compressive and bending strength of phosphogypsum.

**Figure 14:**
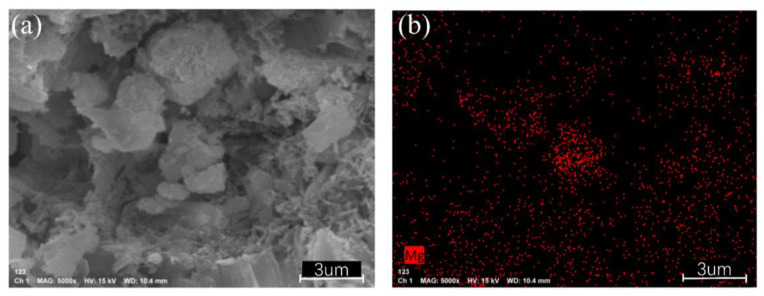
SEM Scanning of Phosphogypsum Specimens and EDS Analysis of Mg; (**a**) SEM Scanning of Phosphogypsum; (**b**) EDS Analysis of Mg.

To clearly observe the position and function of Mg whiskers in phosphogypsum, EDS analysis of Mg elements was conducted on phosphogypsum with added Mg whiskers. The results (as shown in Figure 14) indicate that Mg is primarily distributed in the pores and at the edges of blocky structures. This finding further confirms the functionality of Mg whiskers, which not only fill the pores but also connect the particles, effectively bridging cracks and voids.

## 4. Discussion

This study employed first-principles calculations to investigate the formation energy and mechanical properties of Mg-doped CaSO_4_⋅2H_2_O and calculated the surface energies of CaSO_4_⋅2H_2_O and different magnesium salts, including MgSO_4_, Mg(OH)_2_, and MgCl_2_, on low-index surfaces. Additionally, the stability of interfaces between CaSO_4_⋅2H_2_O/MgSO_4_, CaSO_4_⋅2H_2_O/Mg(OH)_2_, and CaSO_4_⋅2H_2_O/MgCl_2_ was examined. Based on these calculations, magnesium salt whiskers were chosen for doping into pure phosphogypsum. The following conclusions were drawn:

(1) The volumetric modulus, shear modulus, and Young’s modulus of CaSO_4_⋅2H_2_O are 42.52445, 19.76419, and 51.33892 GPa, respectively. After the addition of Mg, these properties increased by 13.43803%, 16.29639%, and 15.62518%, respectively.
(2) Before Mg doping, the Poisson’s ratio of CaSO_4_·2H_2_O is 0.29879, and after doping, it is slightly reduced to 0.29484. The hardness of CaSO_4_·2H_2_O is 3.18363 GPa, while after Mg doping, the hardness of Ca(Mg)SO_4_·2H_2_O reaches 3.6273 GPa, representing a 13.9359% increase in hardness.
(3) The order of interface binding stability between CaSO_4_⋅2H_2_O and different magnesium salts is as follows: CaSO_4_⋅2H_2_O/Mg(OH)_2_ > CaSO_4_⋅2H_2_O/MgSO_4_ > CaSO_4_⋅2H_2_O/MgCl_2_.
(4) After doping magnesium salt whiskers into phosphogypsum, the compressive strength increased by 14.72%; the compressive strength of pure phosphogypsum blocks is 26.35 MPa. After doping with Mg salt, the compressive strength increases to 30.23 MPa, an improvement of 14.72%.

## Figures and Tables

**Table 1:** Composition of Phosphogypsum Blocks.

	CaSO_4_·2H_2_O	Retarder (SG-12P)	Water Reducer (PC-1050)	MSW
PG	Remainder	0.2%	2.3%	—
PG-Mg	Remainder	0.2%	2.3%	0.6%

**Table 2:** C_ij_ (in GPa) of CaSO_4_⋅2H_2_O.${\mathrm{Ca}(\mathrm{Mg})\mathrm{SO}}_{4}\text{·}2H_{2}O$

Species	C_11_	C_12_	C_13_	C_22_	C_23_	C_33_	C_44_	C_55_	C_66_
CaSO_4_⋅2H_2_O	80.94	32.48	29.93	63.40	31.22	56.33	19.60	26.27	20.97
Ca(Mg)SO_4_⋅2H_2_O	88.78	25.61	49.68	59.76	37.52	92.35	25.51	36.37	19.72

**Table 3:** Average Bond Lengths and Population Numbers of CaSO_4_·2H_2_O and Ca(Mg)SO_4_·2H_2_O.

CaSO_4_·2H_2_O					Ca(Mg)SO_4_·2H_2_O				
Bond	$\bar{P}$	$\bar{L}$ (A)	Quantity	Bond	$\bar{P}$	$\bar{L}$ (A)	Quantity	Bond	$\bar{P}$
H-H	−0.0483	2.2855	24	H-H	−0.0459	2.2465	27	H-H	−0.0483
H-O	0.238	1.7114	40	H-O	0.17704	1.9927	54	H-O	0.238
H-S	−0.02	2.8971	8	H-S	−0.0225	2.8879	8	H-S	−0.02
H-Ca	−0.05	2.9642	8	H-Ca	−0.05	2.9542	6	H-Ca	−0.05
			44	H-Mg	−0.11	2.6965	4		
O-O	−0.05	2.8898	16	O-O	−0.1018	2.6427	51	O-O	−0.05
O-S	0.545	1.4991	32	O-S	0.5538	1.4986	16	O-S	0.545

## Data Availability

The original contributions presented in this study are included in the article. Further inquiries can be directed to the corresponding author.
